# Stage III Non–Small-Cell Lung Cancer Treated With Concurrent Chemoradiation Followed or Not by Consolidation Chemotherapy: A Survival Analysis From a Brazilian Multicentric Cohort

**DOI:** 10.1200/JGO.17.00214

**Published:** 2018-05-10

**Authors:** Vladmir C. Cordeiro de Lima, Clarissa S. Baldotto, Carlos H. Barrios, Eldsamira M. Sobrinho, Mauro Zukin, Clarissa Mathias, Facundo Zaffaroni, Rodrigo C. Nery, Gabriel Madeira, Alex V. Amadio, Juliano C. Coelho, Guilherme Geib, Maria Fernanda Simões, Gilberto Castro

**Affiliations:** **Vladmir C. Cordeiro de Lima** and **Rodrigo C. Nery**, AC Camargo Cancer Center; **Clarissa S. Baldotto**, **Mauro Zukin**, and **Gabriel Madeira**, Instituto Nacional de Câncer, Rio de Janeiro; **Carlos H. Barrios** and **Facundo Zaffaroni**, Latin American Cooperative Oncology Group; **Juliano C. Coelho** and **Guilherme Geib**, Hospital de Clínicas de Porto Alegre, Porto Alegre; **Eldsamira M. Sobrinho**, **Clarissa Mathias**, and **Maria Fernanda Simões**, Núcleo de Oncologia da Bahia, Salvador, and **Alex V. Amadio** and **Gilberto Castro Jr**, Instituto de Câncer do Estado de São Paulo, São Paulo, Brazil.

## Abstract

**Purpose:**

Of newly diagnosed patients with non–small-cell lung cancer (NSCLC), stage III accounts for 30%. Most patients are treated with concurrent chemoradiation therapy, but the addition of consolidation chemotherapy (CC) is debatable. We examined the effect of CC in Brazilian patients with stage III NSCLC treated in routine clinical practice.

**Methods:**

We retrospectively collected data for patients from five different Brazilian cancer institutions who had stage III NSCLC and who were treated with chemoradiation therapy followed or not by CC. Eligible patients were age 18 years or older and must have been treated with cisplatin-carboplatin plus etoposide, paclitaxel, or vinorelbine, concurrently with thoracic radiation therapy (RT). Patients treated with surgery or neoadjuvant chemotherapy were excluded. The primary end point was overall survival (OS). Associations between CC and clinical variables and demographics were evaluated by using Pearson’s χ^2^ test. Survival curves were calculated by using the Kaplan-Meier method and were compared using the log-rank test. Univariable and multivariable analysis used a Cox proportional hazards model.

**Results:**

We collected data from 165 patients. Median age was 60 years. Most patients were male (69.1%), white (77.9%), current or former smokers (93.3%), and had stage IIIB disease (52.7%). Adenocarcinoma was the most common histology (47.9%). Weight loss of more than 5% was observed in 39.1% and Eastern Cooperative Oncology Group performance status of 2 was observed in 14.6%. The only variable associated with CC was T stage (*P* = .022). We observed no statistically significant difference in OS between patients treated or not with CC (*P* = .128). A total delivered RT dose ≥ 61 Gy was the only variable independently associated with improved survival (*P* = .012).

**Conclusion:**

Brazilian patients with locally advanced NSCLC who were treated with standard treatment achieved OS similar to that reported in randomized trials. CC did not improve OS in patients with stage III NSCLC after concurrent chemoradiation therapy. An RT dose of less than 61 Gy had a negative effect on OS.

## INTRODUCTION

Lung cancer affected approximately 1.8 million patients worldwide in 2012 and caused 1.6 million deaths.^1^ Non–small-cell lung cancer (NSCLC) accounts for 80% to 85% of lung cancers, and 20% to 35% of these patients will have stage III tumors.^2-4^ Cure rates remain low for those diagnosed with locally advanced (LA) inoperable disease.^1^ For these patients, multimodal therapy has resulted in survival improvement since the introduction of concurrent chemoradiation therapy (CCRT) in the 1990s.^5,6^

More recently, randomized phase III trials have confirmed the superiority of CCRT compared with sequential chemoradiation therapy (CRT). Therefore, the standard of care for LA-NSCLC is CCRT, with a median survival time of approximately 15 months and a 5-year survival rate of 5% to 17%.^7,8^

Various trials have tested whether the addition of consolidation chemotherapy (CC) improved the results of CCRT. Although the results of a phase II trial suggested that CC might improve overall survival (OS), this was not confirmed in subsequent trials.^9,10^

CC is a debatable topic. Although most randomized trials have not demonstrated a survival advantage for the CC approach, it is still frequently used in daily clinical practice. In Brazil, there are no data about the current treatment given to patients with LA-NSCLC or about the efficacy of that treatment.^11^ We decided to assess the effect of CC in Brazilian patients with stage III NSCLC treated in the routine clinical practice scenario in five cancer treatment institutions throughout Brazil.

## METHODS

### Study Design and Population

This study was designed as a multi-institutional retrospective cohort, and information was collected from patients diagnosed with stage IIIA or IIIB LA-NSCLC treated with CCRT between January 2007 to December 2011 in five Brazilian cancer centers (AC Camargo Cancer Center, Instituto Nacional de Câncer, Núcleo de Oncologia da Bahia, Hospital de Clínicas de Porto Alegre, and Instituto do Câncer do Estado de São Paulo). The main objective of the study was to evaluate the effect of the addition of CC on OS. Cancer-specific survival (CSS) and progression-free survival (PFS) were explored as secondary end points.

Data regarding demographics, tumor pathologic features, staging, treatment received, type of response to treatment, and follow-up were recovered from patients’ medical records and registered in a clinical report form specifically designed for this study. No revision of images was undertaken. Staging followed the recommendations of the American Joint Committee on Cancer Guidelines, 7th edition.

For patients to be included in the study, they must have had a histologically confirmed diagnosis of stage IIIA or IIIB LA-NSCLC, have been treated with CCRT, and have received all of their treatment (chemotherapy and radiotherapy) in one of the participating centers. A platinum salt plus etoposide, paclitaxel, or vinorelbine were the only chemotherapy regimens allowed to be given along with radiotherapy (RT) and as CC. Patients were to be excluded if they received CCRT to treat recurrent disease or a second primary, had been diagnosed with another invasive malignant neoplasia ≤ 10 years from the current treatment (in situ carcinoma of the breast or cervix and skin carcinomas were allowed, regardless of the time of diagnosis), had been submitted to surgery with curative intent at any time (except surgical procedures for the purpose of diagnosis or staging), had received brachytherapy or neoadjuvant chemotherapy as part of the treatment plan, had been treated with a chemotherapy regimen different from those mentioned previously, or had participated in any investigational clinical trial.

The study was approved by the local ethics committee at each participating center. A waiver was granted for the necessity of an informed consent because of the retrospective nature of the study. The study was conducted under the auspices of the Grupo Brasileiro de Oncologia Torácica and the Latin American Cooperative Oncology Group.

### End Points

The complete response rate and the objective response rate (complete response rate plus partial response rate) took into account the type of response as recorded in the medical records by the assistant physician. OS was defined as the time between the date of pathologic diagnosis and death as a result of any cause. PFS was defined as the time between the date of pathologic diagnosis and disease progression (locoregional or distant recurrence) or death, whichever occurred first.

### Statistical Analysis

This retrospective cohort did not have a sample size calculation. Descriptive statistics were used to describe numerical and categorical information regarding the patient and disease characteristics. Pearson´s χ^2^ test and adjusted residuals were used to compare categoric variables. Median follow-up time was estimated using the reverse Kaplan-Meier method. We assessed time to event data (PFS, CSS, and OS) by using the Kaplan-Meier product-limit method. The log-rank method was used to compare curves among groups.

Univariable and multivariable analyses were performed by using a Cox proportional hazards model. At univariable analysis, all tested covariates with a significance level ≤ 0.2 were included and tested in the multivariable model. The proportional hazards assumption was tested by using the Schoenfeld residuals technique, and a forward elimination method was used to select the final model (all covariates with significance level ≤ 0.1 were included). All tests were two-sided and performed at the 0.05 significance level. All the analyses were performed using SAS, Version 9.4 (SAS Institute, Cary, NC).

## RESULTS

**Fig 1:**
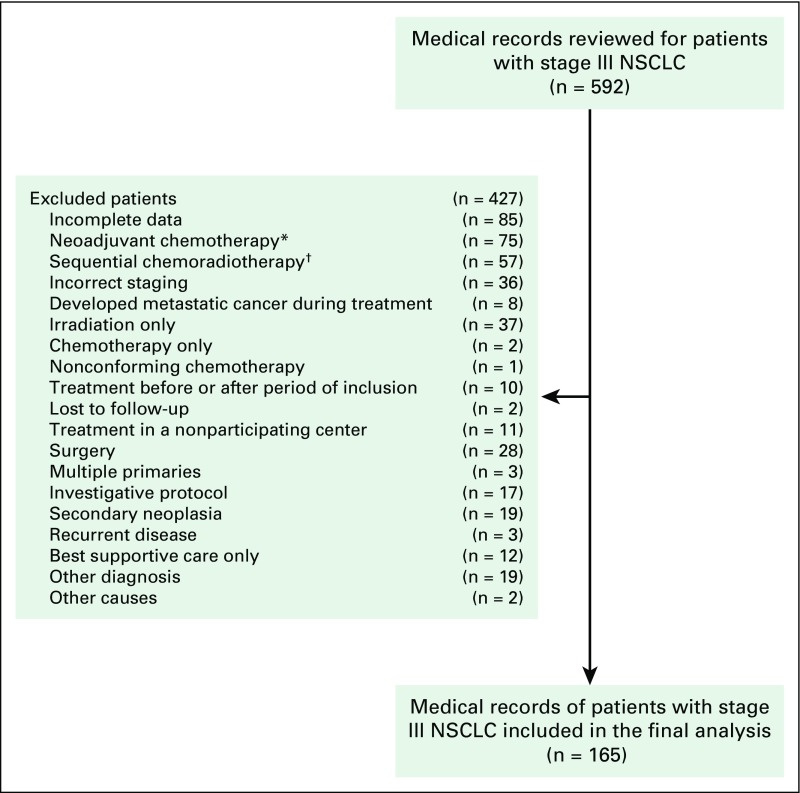
Flowchart with the reasons for exclusion of patients. (*) Induction chemotherapy before chemoradiation. (†) Chemotherapy followed by irradiation only. NSCLC, non–small-cell lung cancer.

We reviewed the medical records of 592 patients diagnosed with stage III NSCLC from five centers that specialized in the treatment of cancer in Brazil. Altogether, data were collected on 165 patients. The main reasons for exclusion were incomplete data in the medical records and the use of neoadjuvant chemotherapy or sequential CRT (Fig 1).

Median age was 60 years (range, 27 to 79 years). Most patients were male (69.1%), white (77.8%), current or former smokers (93.3%), had stage IIIB disease (52.7%), and had a diagnosis of adenocarcinoma (47.9%). Stage T4 was detected in 79 patients (47.9%), and N2 and N3 disease in 95 (57.6%) and 30 patients (18.2%), respectively (Table 1 and Data Supplement). Regarding clinical characteristics, the majority of patients had Eastern Cooperative Oncology Group performance status (ECOG PS) of 1 (75.0%), and most had no weight loss (60.9%) or anemia (75.9%; Table 1).

**Table 1:**
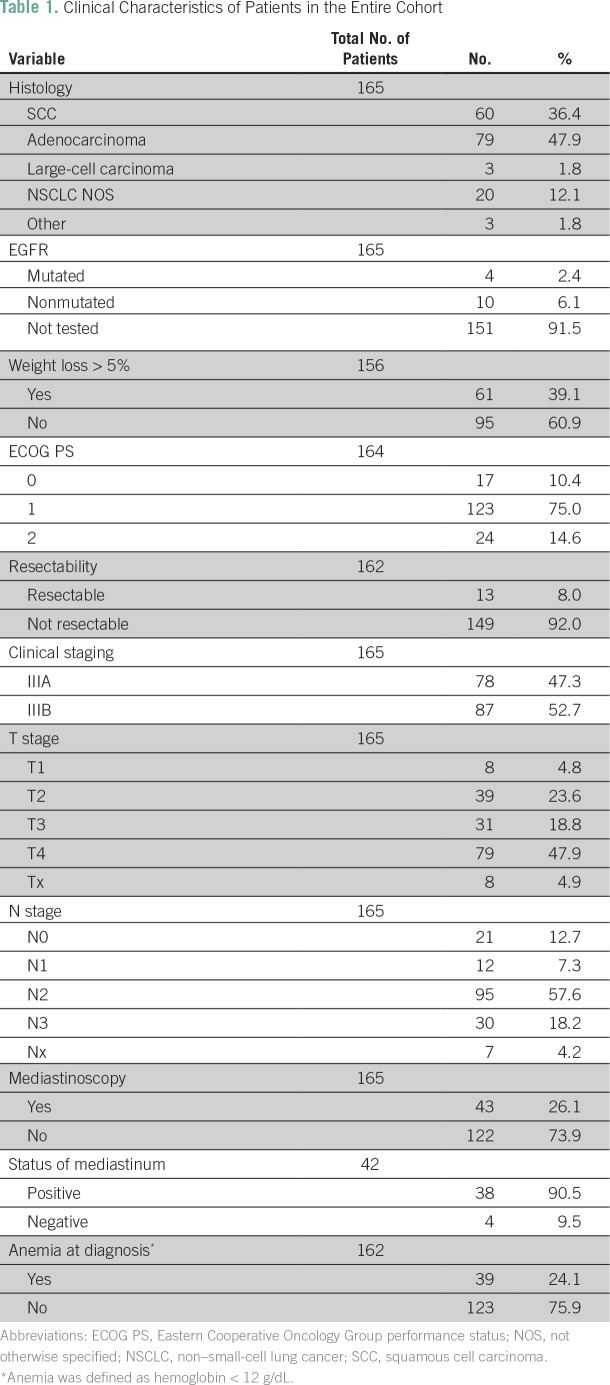
Clinical Characteristics of Patients in the Entire Cohort

CCRT without CC was administered to 83.6% of patients, and the most commonly used chemotherapy schedule was cisplatin plus etoposide, both concurrently with RT (94.5%) and as consolidation (88.9%), followed by carboplatin and paclitaxel (Table 2). Only two patients (1.3%) were treated with intensity-modulated RT (IMRT). RT was interrupted for any reason in 11 patients (8.7%). Objective response rate was achieved in 83 patients (53.2%), complete response was achieved in seven patients (4.5%), and partial response was achieved in 76 patients (48.7%; Data Supplement).

**Table 2:**
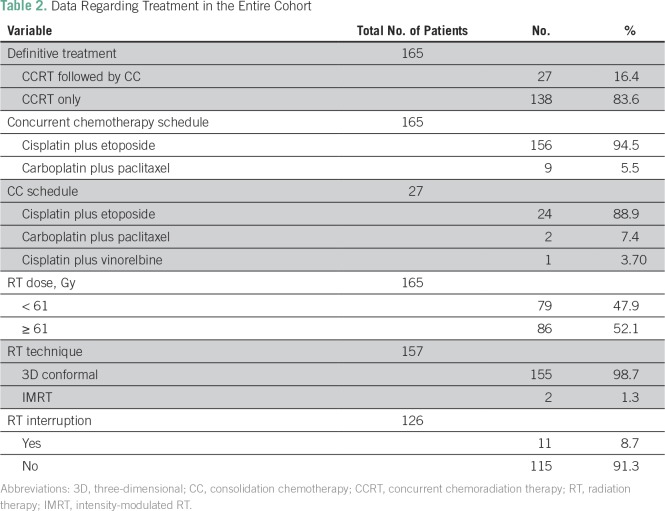
Data Regarding Treatment in the Entire Cohort

The only statistically significant characteristic associated with the administration or not of CC was T stage (χ^2^ = 11.410; *P* = .022). A significant local association (adjusted residual, 2.1) was found relating T3 stage disease and patients who received CC (Data Supplement). Because of this finding, we tested the association of stages T3 and T4 with other clinical or treatment characteristics and found none (Data Supplement). In addition, there was a significant local association (adjusted residual, 2.2) between T2 and no CC.

**Fig 2:**
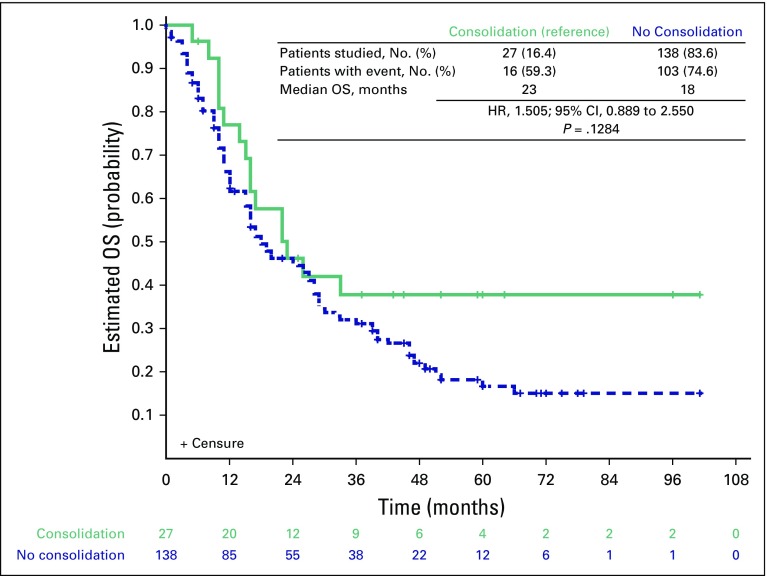
Overall survival (OS) curves according to consolidation chemotherapy status. Survival curves were calculated by using the Kaplan-Meier method and were compared with the log-rank test. Patients still alive were censored at the date of last follow-up.

Median follow-up in the entire cohort was 59 months. Median OS was 19 months (Data Supplement). Estimated OS at 1, 2, and 3 years was 64.8%, 45.5%, and 32.2%, respectively (Data Supplement). On the basis of the log-rank test, the differences in OS between patients who received CC and those who did not were not statistically significant (23 v 18 months; hazard ratio [HR], 1.505; 95% CI, 0.889 to 2.550; *P* = .128; Fig 2). Distant metastasis was the most frequent site of disease progression (55.6%), and cancer was the main cause of death (63.0%).

**Fig 3:**
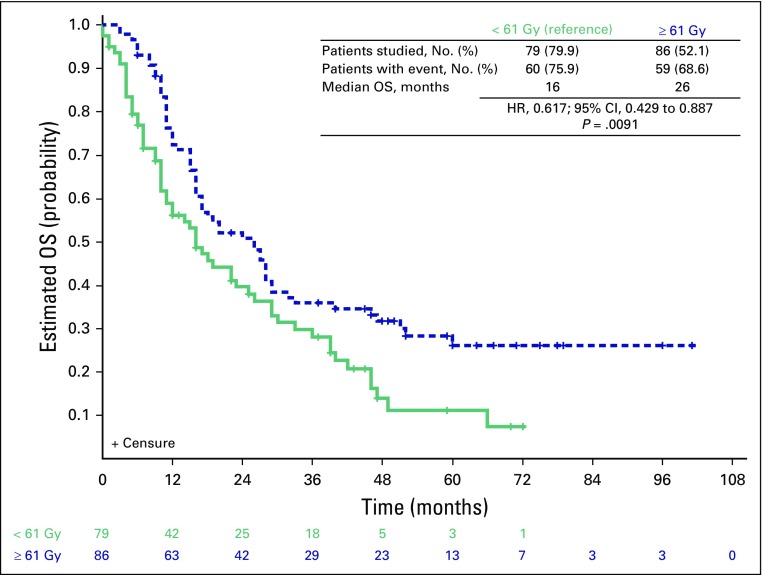
Overall survival (OS) curves according to total irradiation dose. Survival curves were calculated by using the Kaplan-Meier method and were compared with log-rank test. Patients still alive were censored at the date of last follow-up.

Differences in PFS between patients who received CC and those who did not were not statistically significant (HR, 1.451; 95% CI, 0.91 to –2.309; *P* = .1162; Data Supplement). Median PFS was 13 months in the CC group and 9 months in the group with no CC. We did not observe any differences in CSS for patients who received CC or not (23 *v* 26 months; HR, 1.400; 95% CI, 0.810 to 2.421; *P* = .228; Data Supplement). We have not found any association between stage (IIIA or IIIB) and histology (adenocarcinoma or squamous cell carcinoma) with OS, CSS, or PFS (Data Supplement). The only variable independently associated with improved OS was a total RT dose ≥ 61 Gy (16 *v* 26 months; HR, 0.617; 95% CI, 0.419 to 0.909; *P* = .012; Fig 3 and Table 3).

**Table 3:**
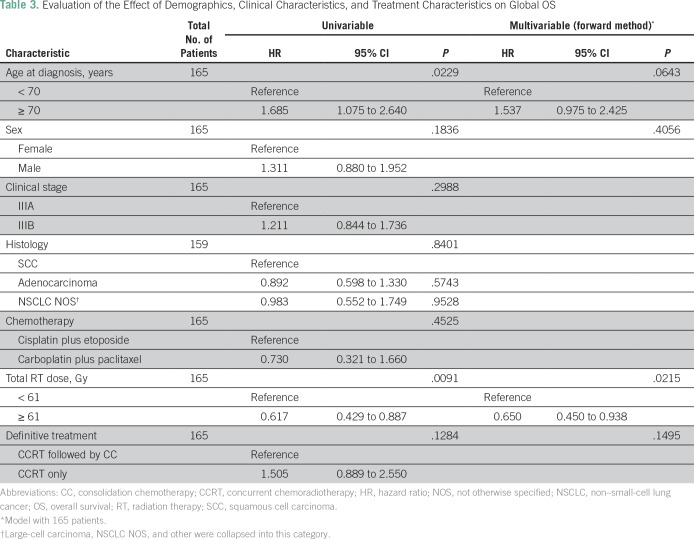
Evaluation of the Effect of Demographics, Clinical Characteristics, and Treatment Characteristics on Global OS

We were able to obtain information about subsequent treatment for 119 patients who had disease progression or recurrence but did not die as a result of other causes. Of these, 73 (61.3%) received some subsequent systemic treatment, mostly cisplatin- or carboplatin-based chemotherapy. Unfortunately, information on the outcome of the treatment was only available for 67 of these patients with a clinical benefit rate of 44.8% (Data Supplement).

## DISCUSSION

To the best of our knowledge, these are the first and only data on the outcome of unselected patients with LA-NSCLC treated with CCRT in Brazil. A previous report described the outcomes of elderly unresectable patients with LA-NSCLC (N = 179) treated between 2003 and 2007 in a single institution in Brazil. In that study, only 29% received CRT (concurrent or sequential), which resulted in improved OS when compared with RT alone or best supportive care, but there was no information about CC.^12^

Interestingly, although we identified a large number of patients with stage III disease (N = 592 [36 patients were incorrectly staged]), most of them did not receive CCRT. Several patients were treated with primary surgery (n = 28) or neoadjuvant chemotherapy (n = 75), probably reflecting a small tumor volume and better PS, or sequential CRT (n = 57) or isolated definitive RT (n = 37), possibly as a result of comorbidities, poor PS, or advanced age.

The retrospective nature of this analysis does not allow a definitive interpretation of the various reasons that justify the selection of approaches that were not standard of care for LA-NSCLC. However, the different treatment alternatives we observed probably reflect the well-known clinical heterogeneity of patients with stage III disease. Of note, these patients were all treated at reference cancer centers where standard CCRT can be routinely administered, but for several undefined reasons, a significant proportion of patients did not receive CCRT. Tentatively, we could argue that, given the recognized heterogeneity of cancer care in Brazil and Latin America, the same, if not a larger proportion of patients with LA-NSCLC treated outside these institutions do not receive what is considered standard therapy. A multicenter prospective collection of data on the management of patients with LA-NSCLC would be interesting and informative.

In 2003, the Southwest Oncology Group SWOG 9504 phase II trial tested the addition of docetaxel as CC after CCRT with etoposide and cisplatin and reported promising results, with a median PFS of 16 months and a median OS of 26 months.^7^ Following that, the Hoosier Oncology Group trial (HOG LUN 01-24) enrolled 243 patients with stage III NSCLC treated with RT and concurrent etoposide and cisplatin to formally test this hypothesis. A total of 166 patients without progression at the end of RT were randomly assigned to three cycles of consolidation with docetaxel or to observation. The median OS was 25 months. No difference was observed between those who received CC with docetaxel and those who did not.^10,13^

In another trial conducted in China and Korea (KCSG_LU05-04), 437 patients were randomly assigned to CCRT alone (once-per-week docetaxel plus cisplatin and concurrent RT to a final dose of 66 Gy) or CCRT followed by three cycles of docetaxel plus cisplatin. There was no statistically significant difference in either PFS or OS.^14^ More recently, a meta-analysis of published data from seven phase III and 34 phase II randomized trials did not show improvement in OS associated with CC after CCRT for patients with LA-NSCLC.^15^ Similarly, the addition of neoadjuvant chemotherapy^16,17^ or neoadjuvant chemotherapy plus CC^18^ to CCRT did not improve OS.

The results in our cohort, which mirrored the results obtained in randomized clinical trials, did not show any improvement in OS with the addition of CC after CCRT, although it should be noted that only 27 patients received CC. Because of the small number of patients in the CC arm, we cannot completely rule out a small difference in favor of CC. Nevertheless, the observed median OS was in the range expected for unresectable stage III NSCLC treated with CCRT. No difference in PFS or CSS was observed, reinforcing the idea that, in general, CC does not enhance outcomes after CCRT.

The vast majority of patients we studied received cisplatin plus etoposide combined in the CCRT schedule (94.5%) and as the CC regimen (88.9%). We did not aim to characterize treatment-related toxicity, but this regimen seemed to be well tolerated because 91.3% of patients did not have any interruption of their RT. Currently, cisplatin plus etoposide and paclitaxel plus carboplatin are both acceptable regimens to be administered concurrently with RT; both seem to be equivalent in terms of efficacy, although the combination of cisplatin and etoposide is associated with more morbidity in real-life.^19^ Another option is the concurrent administration of pemetrexed and cisplatin with RT. In the PROCLAIM study, 598 patients with nonsquamous NSCLC were treated with a pemetrexed plus cisplatin or cisplatin plus etoposide doublet. The enrollment was stopped early because of futility, with no difference in OS between the two arms (26.8 *v* 25.0 months; HR, 0.98; *P* = .831). Nevertheless, a lower incidence of any drug-related grade 3 to 4 adverse events (64.0% *v* 76.8%; *P* = .001) was seen in the pemetrexed plus cisplatin arm.^20^

Although an excess of stage T3 was detected in the group of patients who received CC, it is not likely that this affected the results observed, because no differences in N stage or grouped clinical stage were seen. In addition, clinical stage was not associated with OS in the univariable analysis. The only factor that was independently associated with OS was total dose of RT. Patients treated with a total RT dose of less than 61 Gy experienced decreased median OS (19 months). Koshy et al^21^ have also observed worse OS with total RT doses of less than 59.0 Gy.

The standard dose and fractionation regimen of RT used concomitantly with chemotherapy for stage III NSCLC remains 60 Gy in 30 fractions given once per day. IMRT is preferable to three-dimensional (3D) conformal RT because of the decreased risk of pneumonitis. The dose and method of RT delivery was extensively studied in the RTOG 0617 phase III trial (ClinicalTrials.gov identifier: NCT00533949).^22^ A secondary analysis that compared 3D conformal techniques and IMRT found similar 2-year OS, distant metastasis-free survival, and local failure rates between the two methods of delivery. IMRT was associated with lower heart doses and grade ≤ 3 pneumonitis.^23^ In our study, 3D conformal RT was predominately used instead of IMRT (1.3% of patients), which is a technology that was not widely available at the time. Nevertheless, because of the aforementioned reasons, we believe the selection of patients treated in our study might not have affected the results.

Our study has some limitations. It was retrospective, there was no standard definition of resectability (resectability was defined locally by the surgery team at each participating institution), there was no revision of images to confirm response rate or controlled annotation of toxicity, and the number of patients that received CC was small (which may have reduced our power to detect any significant difference when compared with the number of patients who received CCRT alone). Conversely, patients were treated in cancer treatment centers (all of which used standardized protocols), were distributed among different regions of the country (ensuring adequate geographical representation), and reached survival rates comparable to those observed in randomized trials (pointing to adequate management). Furthermore, there is a large information gap regarding treatment patterns and outcomes of patients with LA-NSCLC in Latin America, especially in Brazil. Thus, this information can help develop and improve local health politics as well as establish and refine local treatment guidelines.

Patients in Brazil who received standard treatment for LA-NSCLC achieved OS and PFS similar to those reported in randomized phase III trials. But a high proportion of patients did not receive standard treatment, probably reflecting stage III heterogeneity and limited access to adequate treatment structure. CC should not be offered to treat LA-NSCLC that is not resectable or is inoperable in common clinical practice, as long as adequate CCRT can be used because it does not improve OS. Efforts should be made to guarantee a minimal total RT dose of 61 Gy concomitantly with chemotherapy.
